# Does different information disclosure on placebo control affect blinding and trial outcomes? A case study of participant information leaflets of randomized placebo-controlled trials of acupuncture

**DOI:** 10.1186/s12874-018-0474-1

**Published:** 2018-01-18

**Authors:** Soyeon Cheon, Hi-Joon Park, Younbyoung Chae, Hyangsook Lee

**Affiliations:** 1 Acupuncture and Meridian Science Research Centre, College of Korean Medicine, Kyung Hee University, Seoul, 02447 South Korea; 2 Department of Public Health, College of Medicine, National Cheng Kung University, Tainan, 70101 Taiwan; 3 Australian Research Centre in Complementary and Integrative Medicine, Faculty of Health, University of Technology Sydney, Sydney, 2007 Australia

**Keywords:** Placebo, Participant information leaflet, Informed consent, Information disclosure, Randomized controlled trial, Blinding, Outcome

## Abstract

**Background:**

While full disclosure of information on placebo control in participant information leaflets (PILs) in a clinical trial is ethically required during informed consent, there have been concerning voices such complete disclosures may increase unnecessary nocebo responses, breach double-blind designs, and/or affect direction of trial outcomes. Taking an example of acupuncture studies, we aimed to examine what participants are told about placebo controls in randomized, placebo-controlled trials, and how it may affect blinding and trial outcomes.

**Methods:**

Authors of published randomized, placebo-controlled trials of acupuncture were identified from PubMed search and invited to provide PILs for their trials. The collected PILs were subjected to content analysis and categorized based on degree of information disclosure on placebo. Blinding index (BI) as a chance-corrected measurement of blinding was calculated and its association with different information disclosure was examined. The impact of different information disclosure from PILs on primary outcomes was estimated using a random effects model.

**Results:**

In 65 collected PILs, approximately 57% of trials fully informed the participants of placebo control, i.e. full disclosure, while the rest gave deceitful or no information on placebo, i.e. no disclosure. Placebo groups in the studies with no disclosure tended to make more opposite guesses on the type of received intervention than those with disclosure, which may reflect wishful thinking (BI −0.21 vs. −0.16; *p* = 0.38). In outcome analysis, studies with no disclosure significantly favored acupuncture than those with full disclosure (standardized mean difference − 0.43 vs. −0.12; *p* = 0.03), probably due to enhanced expectations.

**Conclusions:**

How participants are told about placebos can be another potential factor that may influence participant blinding and study outcomes by possibly modulating patient expectation. As we have few empirical findings on this issue, future studies are needed to determine whether the present findings are relevant to other medical disciplines and at the same time a routine practice of fully disclosing placebo information in PILs calls for reevaluation.

**Electronic supplementary material:**

The online version of this article (10.1186/s12874-018-0474-1) contains supplementary material, which is available to authorized users.

## Background

Informed consent is a process in clinical research to ensure participants’ autonomy is respected, and a document called participant information leaflet (PIL) is often used to assist the informed decision making process for the participants by providing adequate information. Potential trial participants are given thorough information such as study aims, methods, anticipated benefits and potential risks of participation from PILs [1].

Use of a placebo control is often implemented in randomized controlled trials (RCTs) not only to evaluate the specific effect of a test intervention but also to effectively blind study participants. Unlike trials of pharmacological interventions where fabrication of placebo is relatively easy, however, inventing and/or implementing a placebo control and ensuring masking in trials assessing non-pharmacological treatments such as surgery, psychotherapy, or acupuncture, is known to be more difficult [2]. In non-pharmacological trials, it has been challenging since detailed information on study hypothesis may compromise the success of blinding while ensuring participant’s autonomy is an essential ethical research conduct. Placebo-controlled trials of acupuncture have been in particular criticized for being stingy with placebo information disclosure to their participants [3, 4]. Such practice, i.e. partial or no information on study hypothesis and methods to the study participants, is line with one of the strategies used in studies assessing non-pharmacological treatment to achieve successful blinding [2].

Considering the accumulating evidence supporting that what information is given to participants before trial can affect their expectation and behavior both in positive (i.e. placebo effect) and negative (i.e. nocebo effect) ways [5–8], and that at the same time, success of blinding ensures minimizing risk of bias in a trial, the importance of information in PILs cannot be disregarded. Moreover, unlike PILs from conventional drug trials that accurately disclose the use of placebo [9], PILs of randomized placebo-controlled trials of acupuncture seem to vary in the way they describe placebo controls [3]. There have been few empirical studies examining PILs of randomized placebo-controlled trials [3], and to our knowledge, the link between placebo information in PILs and results of blinding success or study outcomes has rarely been explored.

In this context, the present study collected PILs of randomized placebo-controlled trials of acupuncture 1) to identify what trial participants are told about placebo controls, and further analyzed 2) whether the information on placebo acupuncture procedures from collected PILs affect the direction/degree of participant blinding and the trial outcomes.

## Methods

This study was approved by the Kyung Hee University Ethics Committee (KHSIRB-13-052 (EA)).

### Study inclusion and PIL collection

PubMed was searched from January 2011 to November 2013 to identify RCTs of manual acupuncture, electroacupuncture, and laser acupuncture where sham or placebo device or procedure was used for control group. No language restriction was imposed. For the RCTs predating 2011, we adopted a list of included studies in another systematic review of randomized, placebo-controlled trials of acupuncture with blinding evaluation data, published in English between 1985 and 2011 [10].

When the list of eligible studies was completed, first or corresponding authors were invited via e-mail to send in PILs or informed consent forms used in their trials. If the authors refused to provide their PILs, or there was no response after the second reminder, studies were consequently excluded from our analysis. PILs in languages other than English were translated into English by native language speaker.

### Content analysis of PILs

Through iterative reading of PILs and following steps of systematic methods developed for deductive content analysis [11–13], we reached a consensus of final three categories by description/explanation of placebo acupuncture: “full disclosure of placebo acupuncture (FD)”, “deceptive disclosure of placebo acupuncture (DD)”, and “missing information on placebo acupuncture (MI)”. The PILs had to use specific words such as ‘sham’, ‘placebo’, or ‘fake’ acupuncture, to be coded in FD category. PILs that used neutral or deceitful words like ‘group two’, or simply the ‘control group’ were put into DD category even though we trusted that the study authors never intended to be ‘deceptive’ on purpose in their PILs. However, if they were followed by additional explanation suggesting that neutrally/deceitfully named group would involve sham procedures (e.g. to receive non-acupoint acupuncture), it was considered to be in the FD category since it was judged that sufficient information was given to the study participants to perceive the existence of placebo acupuncture. For trials with more than one control groups applying different types of placebo acupuncture, each placebo group was counted as a separate unit of data. PILs that missed out on explaining anything about a placebo control were coded into MI category.

### Information on placebo and blinding

From studies providing results on success of blinding, Bang’s blinding index (BI) [14] was calculated with 95% confidence intervals (CI) to further investigate the association between information on placebo and effectiveness of blinding in trials. Bang’s BI yields a value between − 1 and 1 for each arm in a study, providing the proportion of the potentially unblinded beyond chance [14]. BI of 1 would mean that everyone guessed correctly, and 0 means guesses were random, whereas − 1 would mean that all guesses were incorrect (e.g. all participants in the placebo arm answered that they believe they received real treatment). The number of participants in each response of each arm was extracted from the included studies and more information was sought from the original authors if necessary. If a study measured outcome at more than one time point, the data measured at the end of the last intervention or at the closest were extracted. Based on BIs, each study was classified into one out of nine possible blinding scenarios: in brief, random guess, unblinded or opposite guess is given to each arm in a study, and then, nine blinding scenarios are possible [10, 14, 15]. The threshold for blinding classification was random guess for − 0.2 < BI < 0.2; unblinded for BI ≥ 0.2; and opposite guess for BI ≤ − 0.2 [10]. It should be noted that throughout this paper, the term “unblinded” generally means “more correct guess,” not broken blinding literally.

Assigned scenarios and calculated BIs were compared between “disclosure (FD) and no disclosure (DD/MI)” groups using Mann-Whitney U test or independent t-test depending on the data distribution by Shapiro-Wilk test using SPSS (IBM Corp., Version 21.0, Armonk, NY), and the statistical significance was set at *p* < 0.05.

### Information on placebo and trial outcomes

The aim of this meta-analysis was not to estimate the effects of acupuncture, but to synthesize the primary outcomes so that the summarised effects of studies from disclosure and no disclosure groups can be compared. For this analysis, following strategies were used to choose the outcome: (1) whether it is continuous outcome or dichotomous outcome, it should have data that are needed to calculate standardized mean difference (SMD) and standard deviation (SD); (2) the timing should be either indicated primary end-point or as close as possible to the last treatment session; (3) in the absence of predefined single main outcome measure, commonly used outcome in similar studies (e.g. visual analogue scale (VAS) for pain studies) was chosen or; (4) an outcome that reports clear data (e.g. if one outcome is reported in a table with specific values whereas another outcome is reported in a figure without any accurate values, the former) was chosen; (5) if a trial had more than one placebo group, the data were pooled separately as they were in content analysis, and number of participants in the shared intervention group was divided evenly [16]. For calculating SMD and SD from dichotomous outcomes, we used a formula, $\mathrm{SMD}=\frac{\sqrt{3}}{\pi}\ln OR$(OR, odds ratio) [17]. A random-effects model with the generic inverse variance method was adopted to estimate SMD and 95% CI using Review Manager (The Nordic Cochrane Centre, Version 5.3.4, Copenhagen, Denmark). The *I*^2^ test was used to examine heterogeneity across the studies, and different PIL categories were subject to the subgroup analysis to investigate whether the estimates in the primary outcome varied between subgroups (FD or DD). In this meta-analysis using a Review Manager software, this test was undertaken using Cochran’s Q test and Higgins’ *I*^2^ by comparing the subtotal estimates between the subgroups. In addition, a post hoc analysis grouped studies by its originating region (Asia and Non-Asia) in each PIL category and tested in the same manner above whether the estimates in the outcome of interest varied in different regions.

## Results

### Included PILs

The initial PubMed search yielded 216 records. Among them, 107 articles were excluded for not meeting our inclusion criteria: studies with no acupuncture group per inclusion criteria (*n* = 39), non-RCTs (*n* = 26), protocols only (*n* = 20), studies with no placebo acupuncture control group (*n* = 19), animal studies (*n* = 2), and a duplicate (*n* = 1) [18, 19]. Additional 53 studies included in a previous systematic review [10] of placebo-controlled trials of acupuncture were then added to the remaining 109 studies. One study [20] from the adopted list was replaced by its original trial as it was identified as an additional report [21]. Two studies overlapping with our own search result were excluded, leaving a total of final 160 studies subject to invitation to share PILs.

**Fig. 1:**
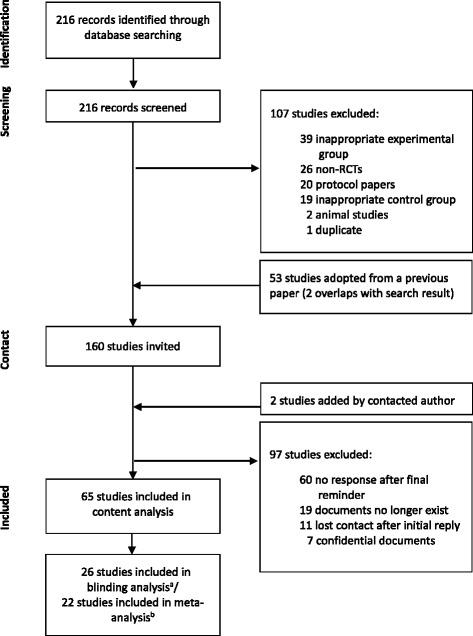
Flow diagram for selection of studies. ^a^Among included studies, only those providing extractable data for calculating blinding index were included here. ^b^Studies that clearly indicated and reported specific data of primary outcome were eligible for meta-analysis. RCT, randomized controlled trial

The invitation e-mail was sent to the trialists in 24 countries in total, with China and USA on the top with 29 trials followed by Germany, Korea, and UK. Among 100 studies that replied to our invitation letter (62.5% of response rate), 63 studies provided PILs. In addition, one author generously provided two extra PILs on top of the requested documents and final 65 PILs from 15 different countries were collected for analysis (Fig. 1; Additional file 1: Appendix 1).

### Placebo control groups and information disclosure in PILs

Types of placebo acupuncture implemented in the included studies were diverse and could be simply divided into three groups; non-penetrating placebo acupuncture, penetrating placebo acupuncture, and others (Additional file 1: Appendices 2 and 3).

Through the content analysis of information on placebo given to study participants, extracted data of 70 placebo control groups from 65 PILs were categorized into three groups. More than half of the PILs (57.1%) were straightforward to the participants when they were introducing placebo acupuncture control, and these were classified as FD group. In the 2nd group named as DD group, no words or phrases that clearly indicated placebo acupuncture would be used (35.7%), i.e., rather deceptive words such as ‘different (7),’ ‘control (5),’ ‘group 1 or group 2 (2),’ ‘non-traditional (2),’ ‘not typical (2),’ and ‘test (2),’ were given. Remaining five PILs (7.1%) fell into MI group and they missed out on explaining anything about a placebo control group. Detailed results of the content analysis are provided in Additional file 1: Appendix 4.

Our analysis of PILs by country of its origin revealed that PILs from non-Asian countries were more likely to fully inform the study participants of placebo acupuncture compared with those from Asian countries (χ^2^ (1, *n* = 70) = 7.099, *p* = 0.008; Additional file 1: Appendix 5).

### Information on placebo and blinding

Data for BI calculation were available in 26 studies (Fig. 1). With one study providing three independent data sets [22], 28 sets of BI were calculated in total. Among the nine possible scenarios of blinding (Table 1), 50% of the studies (*n* = 14) were counted into blinding scenarios 1 (random guess for both acupuncture and control groups) and 5 (unblinded for acupuncture group and opposite guess for control group), which are assumed to be the ideal blinding situation. In the scenarios considered possibly problematic, which are blinding scenarios 4 (unblinded for both acupuncture and control groups) and 6 (unblinded for acupuncture group and random guess for control group), 35.7% of the studies (*n* = 10) were included. When the number of studies that belonged to ideal or possibly problematic blinding scenarios was compared within each category, DD/MI category had a higher rate of studies with ideal blinding than FD category (Table 1). The number of studies in S1 and S5, the ideal scenarios, was more than double the number of studies in S4 and S6 in DD/MI category (7 studies vs. 3 studies), whereas the numbers were the same in FD category (5 studies vs. 5 studies).

**Table 1:** Frequencies of nine different blinding scenarios by PIL categories

Scenario	Experimental Group	Control Group	Possible Interpretation [10]	FD % (n)	DD/MI % (n)
S1	Random guess	Random guess	Possibly most ideal from the scientific or statistical perspective	11.8 (2)	18.2 (2)
S2	Random guess	Opposite guess	Rare	5.9 (1)	9.1 (1)
S3	Random guess	Unblinded	Possibly little treatment effect and no effect in control group	5.9 (1)	0 (0)
S4	Unblinded	Unblinded	Possibly problematic	11.8 (2)	9.1 (1)^a^
S5	Unblinded	Opposite guess	Ideal – patients tend to have wishful thinking, strong placebo effect, and any treatment administered is perceived as real treatment	29.4 (5)	45.5 (5)
S6	Unblinded	Random guess	Possibly problematic – patients in control group do not know what to expect in the absence of treatment	29.4 (5)	18.2 (2)
S7	Opposite guess	Opposite guess	Rare	0 (0)	0 (0)
S8	Opposite guess	Random guess	Rare	5.9 (1)	0 (0)
S9	Opposite guess	Unblinded	No treatment effect at all or patients may have low expectations	0 (0)	0 (0)

There were 17 and 11 studies in FD, and DD/MI category, respectively. ^a^Among the 11 studies in DD/MI category, only one article belonged to MI category

Here, the term “Unblinded” generally means “More correct guess,” not broken blinding literally

Random guess: −0.2 < BI < 0.2; unblinded: BI ≥ 0.2; opposite guess: BI ≤ −0.2 [10]

*BI* Blinding index, *DD* Deceptive disclosure of placebo acupuncture, *FD* Full disclosure of placebo acupuncture, *MI* Missing information on placebo acupuncture, *PIL* participant information leaflet

**Fig. 2:**
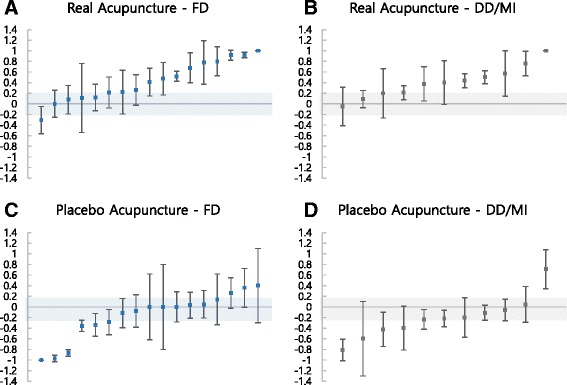
BI values by PIL categories. **a** BI values of real acupuncture group from FD category; **b** BI values of real acupuncture group from DD/MI category; **c** BI values of placebo acupuncture group from FD category; **d** BI values of placebo acupuncture group from DD/MI category. Negative values indicate opposite guessing of allocated arm, 0 refers to random guessing, and positive values indicate correct guessing of allocated arm (e.g. guessed real acupuncture when assigned to real acupuncture). This study defined BI value between − 0.2 and 0.2, presented in the shaded area in each graph, as random guess [10]. BI, blinding index; DD, deceptive disclosure of placebo acupuncture; FD, full disclosure of placebo acupuncture; MI, missing information on placebo acupuncture; PIL, participant information leaflet

BI values for each study were calculated with their 95% CI (Fig. 2). Average BI values for real acupuncture groups in FD and DD/MI categories were 0.42 and 0.41, respectively. In other words, 42% (95% CI 0.23 to 0.62) of participants in the real acupuncture groups who were given full disclosure of placebo information and 41% (95% CI 0.21 to 0.61) in the real acupuncture groups from the studies with no disclosure (DD/MI category) correctly guessed that the given treatment was real acupuncture beyond chance (Fig. 2a and b). On the other hand, 16% of the participants in the placebo group in the studies with full information disclosure believed that they received real acupuncture beyond chance (− 0.16, 95% CI −0.39 to 0.06, Fig. 2c). In the groups without adequate information on placebo acupuncture, 21% of the participants in the placebo group guessed they were given real acupuncture (− 0.21, 95% CI −0.47 to 0.05, Fig. 2d). There was no significant difference in BI values of both real and placebo acupuncture groups between the two categories, i.e. disclosure vs. no disclosure (real acupuncture, *p* = 0.92, independent *t*-test; placebo acupuncture, *p* = 0.38, Mann-Whitney *U* test).

### Information on placebo and trial outcomes

**Fig. 3:**
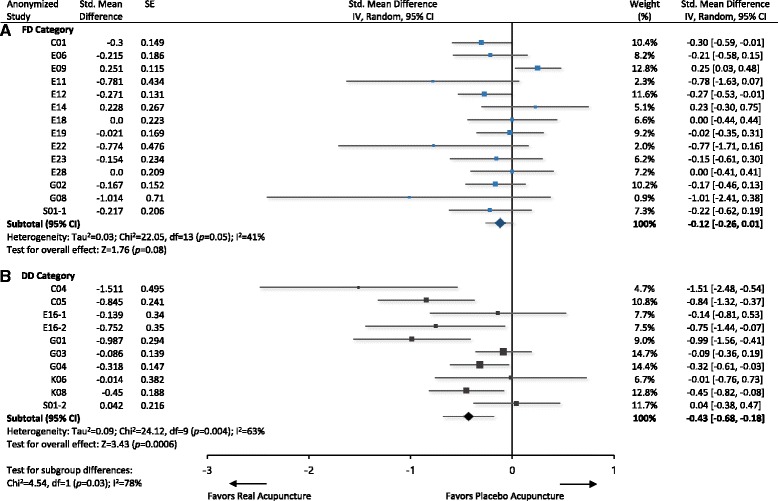
Clinical outcomes by PIL categories. Anonymized studies are from (**a**) FD category, and (**b**) DD category, respectively. A test for subgroup differences indicates that studies from DD category report greater effects of real acupuncture compared to those from FD category. CI, confidence intervals; DD, deceptive disclosure of placebo acupuncture; FD, full disclosure of placebo acupuncture; PIL, participant information leaflet; SE, standard error; Std., standardized

Among 22 studies of which SMD and SD were available, two studies had two placebo control groups, leaving 24 units of data for a meta-analysis. No study from MI category provided adequate data for pooling. The meta-analysis of studies in DD category showed acupuncture produced better clinical outcomes compared to placebo acupuncture (SMD −0.43, 95% CI −0.68 to − 0.18, *p* = 0.0006; Fig. 3), whereas the studies of FD category found no significant difference between real and placebo groups (SMD −0.12, 95% CI −0.26 to 0.01, *p* = 0.08; Fig. 3). When two categories were assigned as subgroups, a test for subgroup differences also showed that pooled effect for real acupuncture from DD category is greater than that of FD category (χ^2^ = 4.54, *p* = 0.03, I^2^ = 78.0%; Fig. 3).

A post hoc subgroup analysis was performed within each category to see if there is any regional difference in trial results, but this analysis could not confirm the results of Vickers et al. [23] which found Asian countries were more likely to produce positive findings in acupuncture studies. In both categories, there was no difference between studies from Asian and non-Asian countries (test for subgroup differences; FD, *p* = 0.88; DD, *p* = 0.23; Additional file 1: Appendix 6).

## Discussion

### Principal findings of the study

We analyzed PILs of placebo-controlled acupuncture trials to see how placebo acupuncture was described to the study participants. Approximately 57% of the studies fully informed the participants of placebo acupuncture while the rest of the studies either gave deceitful or no information on placebo. A half of the studies were evaluated as ideally blinded. When BI values of the included studies were compared between FD and no disclosure (DD/MI) categories, placebo groups in the latter were more likely than those in FD category to make opposite guesses on the intervention, i.e., guessed that they have received real acupuncture. Although this difference did not reach statistical significance, it may reflect more wishful thinking of placebo groups in no disclosure category than those in FD category. Studies with no disclosure reported greater treatment effects for real acupuncture than those with FD, possibly due to enhanced expectations.

### Possible explanations and implications of the study for clinicians and researchers

In medicine, whether in clinical or research settings, there are high levels of ethical requirements that are expected to be met. Respecting trial participants’ autonomy by providing them adequate amount of information is one of those requisites for ethical practice in medical research, and often, the PIL is presented to the participants as a tool to meet the requirements. The main purpose of the PIL, in this sense, would be to help participants voluntarily make a well-informed decision on the introduced matter. When the control intervention of the trial involves a placebo, there has to be even more cautious approach and full disclosure of such information [1, 24, 25]. On the other hand, there are concerns on too much information may disadvantage the participants, as in increasing a nocebo effect [26, 27], or resulting in a lengthy document that rather compromises participants’ understanding [28]. Some argue that PILs are not in a reader friendly language [29], nor come with decision making aids [30]. Therefore, for a PIL to fully serve its purpose, it seems necessary to inform the participants in a way that is not only being clear about what is going to happen, but also being considerate of how it actually helps them to make an informed decision.

There has been a concerning voice that placebo-controlled trials of acupuncture are reluctant to fully disclose the existence of placebo acupuncture control [3, 4]. However, findings of the current study show change of practice ever since, showing number of studies that accurately inform the use of placebo control, being more than a half: out of 70 placebo groups from 65 PILs, 40 were classified into FD category, and 12 of those 40 were from the older studies that were included in the previous review. These findings confirm that acupuncture trials are also trying to conform to the ethical requirements [31, 32]. However, at the same time, given that there is still a room for improvement in providing complete and accurate information about placebos even in trials assessing conventional medicine [9], and device-based interventions are facing challenges in maintaining blinding with a full disclosure of placebos [33], information disclosure in PIL may be taken as an issue in clinical research across the board.

In East Asian countries, even after western medicine’s addition to the system, traditional medicine is still routinely used [34, 35]. As a result, acupuncture is easily accessible and embedded in cultural background. Therefore, participants as well as the researchers of acupuncture trials are assumed to be more familiar with acupuncture compared to those in non-Asia region [36]. This brings concern to Asian researchers that the study participants will easily distinguish real from placebo acupuncture. The rationale behind such fear is not simple, as studies examining the influence of the participant’s previous experience on detecting placebo acupuncture presented conflicting results [37, 38]. The notion that blinding may be hampered if trial participants are fully informed on details of placebo acupuncture, is partly explained in our BI analysis. While 21% of the participants in the placebo acupuncture group from DD/MI category made incorrect guesses beyond chance, i.e. they believed they received real acupuncture, 16% of those in the placebo acupuncture group from FD category made such guesses. It could be interpreted that though not statistically significant, the participants on the placebo acupuncture arm who were not very well informed about placebo acupuncture tended to guess that they received real acupuncture more than participants in FD category did. It reflects participants’ wishful thinking, which may lead to an augmented placebo effect [10]. This finding is in line with the result from the Cochrane review of all placebo interventions where a higher placebo effect was reported in trials without correct information of placebo than those with correct information [39]. An altered placebo labeling study also has shown that placebo effect goes higher as the label on placebo changes from placebo, uncertain to valid medicine [40]. Given our BI analysis and the results from other relevant studies, the hypothesis can be generated that full disclosure of placebo in PILs may undermine successful blinding of participants and this should be systematically evaluated in the future studies.

A meta-epidemiological study has reported that well maintained blinding resulted in a smaller effect of intervention compared to non-blinded trials [41]. In the present study, however, pooled effect size of trials in DD category, which showed better blinding results than FD category, was greater than that of FD category. There could be two possible explanations. First, we may suspect that informing the presence of placebo acupuncture in detail lowered the participants’ expectation and as a result, possibly undermined the effect of real acupuncture in studies in FD category, whereas the participants’ expectation was maintained or enhanced in DD category and the trials favored acupuncture to a larger extent. This is supported by a pooled analysis from four, large population based German RCTs which demonstrated higher expectation was associated with a better outcome regardless of given treatment [42]. Considering that our study was not designed to directly measure participants’ expectation of treatment, however, we cannot estimate to what extent expectation contributed to greater effect size from the studies in DD category than those in FD category. Another explanation is that the treatment effect in DD category is rather overestimated due to investigator’s bias. Not only the patients but also trialists have expectations, wittingly or unwittingly [43]. Considering the fact that more studies in DD/MI category originated from Asia, which is an acupuncture friendly region where researchers are eager to prove its efficacy, than FD category, it may be trialists’ expectation on top of the participants’ expectation that resulted in such higher estimation. Besides, when we evaluated whether outcome assessor was blinded to the intervention in the included studies, the proportion of studies with appropriate blinding for outcome assessor was slightly lower in DD category (20 out of 25, 80%) compared to FD category (33 out of 40, 82.5%). This too supports investigator bias may have possibly played a part in overestimation of treatment effect in the studies of DD category. Nonetheless, the fact that only small number of studies were included here and that this is merely observational in nature allows for an interpretation in a narrow context. To elucidate complicated relationships between PILs, blinding, and outcomes of a trial, further studies designed to compare the links between those are warranted for more discussion.

### Strengths and weaknesses of the study

One of the strengths of this study is that we are addressing a fundamental question over so-called routine clinical research procedures. We tried to provide a more comprehensive understanding of any association of placebo disclosure in PILs with methodological issues in clinical research, i.e. blinding, and the effect size. Previous studies have usually focused on ethics [44], readability [45], or comprehensiveness [46], but few studies explored association of pre-trial information on placebo with blinding and trial outcomes, which definitely requires further investigations from a different point of view. Another strength would be that it encompasses qualitative and quantitative research designs. Collected PILs were subject to systematic qualitative content analysis method [12]. Then, numerical analyses such as calculating BI and effect size were conducted in conjunction with the results from content analysis. Such integrated method can help science achieve better understanding of its discoveries [47].

Some limitations also should be noted. Firstly, not all included documents were written in English and simple analysis such as word count, page count, or readability calculation was not feasible. Although, all documents were carefully translated into proper English by experts then analyzed in the main analysis in a standardized way, we cannot rule out the possibility that some subtle nuance of original language was not delivered in translation. Secondly, the number of studies included in blinding and outcome analysis was too small compared to that of all included studies for content analysis, which might have made it difficult to detect statistically significant differences, if any, thus to draw any firm conclusion. Even so, the number of studies included in the main content analysis was larger than 16 of a previous study [3] and was similar to the suggested ‘medium’ sample size for integrated qualitative-quantitative research [47]. More importantly, this study accomplished to collect the documents from many different countries of different environment and culture while the previous study had documents mostly from only few countries such as Germany, UK and USA. This diversity of documents in the present study made it possible to explore rich source of data. Last but not the least, the actual perception of the information on PIL by study participants may differ from degree of information disclosure judged in our study. Participants’ understanding and memory of the contents of PILs vary [33] and their behavior may be influenced by other multiple factors such as prior knowledge, health literacy, preference and expectation.

### Unanswered questions and future research

Our findings raise questions on routinely accepted informed consent procedures: ethical standard requires every detailed information including placebo should be given to study participants. Placebo information in other than acupuncture trials, however, has been reported far from fully informing [9] and what information is provided to the patient about side effects is known to modulate treatment responses often to enhance nocebo effect [8, 48]. Moreover, as we have few empirical findings on how patients are told about placebo may affect blinding and study outcomes, future studies are needed to examine this unanswered question.

Other than methodological qualities such as allocation concealment or blinding that are known to affect the direction or magnitude of treatment effect [41, 49], we found information on placebo in PIL may be another potential factor for influencing the study outcome by possibly modulating patient expectation in the informed consent process. Therefore, further in-depth studies utilizing both qualitative and quantitative methods are warranted to better understand complicatedly intertwined factors in PILs, blinding, and outcomes of placebo-controlled trials.

## Conclusions

How participants are told about placebos may be another potential factor that may influence participant blinding and the study outcome. As we have few empirical findings on this issue, future studies are needed to determine whether the present findings are relevant to other medical disciplines and at the same time a routine practice of fully disclosing placebo information in PILs calls for reevaluation.

## Additional file

Additional file 1: **Appendix 1.** Demographics of collected PILs. **Appendix 2.** Placebo control groups in the included studies. **Appendix 3.** Characteristics of the included studies. **Appendix 4.** Description of placebo acupunctures in PILs. **Appendix 5.** How different regions describe placebo acupuncture differently. **Appendix 6.** A post-hoc subgroup analysis between studies from Asian and non-Asian countries. (DOCX 3144 kb)

## Abbreviations

BI: Blinding index
CI: Confidence interval
DD: Deceptive disclosure
FD: Full disclosure
MI: Missing information
OR: Odds ratio
PIL: Participant information leaflet
RCT: Randomized controlled trial
SD: Standard deviation
SMD: Standardized mean difference

## References

[1] World Medical Association (2013). World medical association declaration of Helsinki: ethical principles for medical research involving human subjects. JAMA.

[2] Boutron I, Guittet L, Estellat C, Moher D, Hróbjartsson A, Ravaud P (2007). Reporting methods of blinding in randomized trials assessing nonpharmacological treatments. PLoS Med.

[3] Linde K, Dincer F (2004). How informed is consent in sham-controlled trials of acupuncture?. J Altern Complement Med.

[4] Miller FG, Kaptchuk TJ (2007). Acupuncture trials and informed consent. J Med Ethics.

[5] Miller FG, Colloca L (2011). The placebo phenomenon and medical ethics: rethinking the relationship between informed consent and risk–benefit assessment. Theor Med Bioeth.

[6] Tracey I (2010). Getting the pain you expect: mechanisms of placebo, nocebo and reappraisal effects in humans. Nat Med.

[7] Carlino E, Torta DM, Piedimonte A, Frisaldi E, Vighetti S, Benedetti F (2015). Role of explicit verbal information in conditioned analgesia. Eur J Pain.

[8] Mitsikostas DD (2016). Nocebo in headache. Curr Opin Neurol.

[9] Bishop FL, Adams AE, Kaptchuk TJ, Lewith GT (2012). Informed consent and placebo effects: a content analysis of information leaflets to identify what clinical trial participants are told about placebos. PLoS One.

[10] Moroz A, Freed B, Tiedemann L, Bang H, Howell M, Park JJ (2013). Blinding measured: a systematic review of randomized controlled trials of acupuncture. Evid Based Complement Altern Med.

[11] Finfgeld-Connett D (2014). Use of content analysis to conduct knowledge-building and theory-generating qualitative systematic reviews. Qual Res.

[12] Hsieh HF, Shannon SE (2005). Three approaches to qualitative content analysis. Qual Health Res.

[13] Mayring P. Qualitative content analysis. Forum Qual Soc Res. 2000;1(2) 10.17169/fqs-1.2.1089.

[14] Bang H, Ni L, Davis CE (2004). Assessment of blinding in clinical trials. Control Clin Trials.

[15] Park J, Bang H, Cañette I (2008). Blinding in clinical trials, time to do it better. Complement Ther Med.

[16] Higgins JPT, Deeks JJ, Altman DG, Higgins JPT, Green S (2011). Chapter 16: special topics in statistics. Cochrane handbook for systematic reviews of interventions.

[17] Chinn S (2000). A simple method for converting an odds ratio to effect size for use in meta-analysis. Stat Med.

[18] Wang LP, Zhang XZ, Guo J, Liu HL, Zhang Y, Liu CZ (2012). Efficacy of acupuncture for acute migraine attack: a multicenter single blinded, randomized controlled trial. Pain Med.

[19] Wang LP, Zhang XZ, Guo J, Liu HL, Zhang Y, Liu CZ (2011). Efficacy of acupuncture for migraine prophylaxis: a single-blinded, double-dummy, randomized controlled trial. Pain.

[20] Kaptchuk TJ, Kelley JM, Conboy LA, Davis RB, Kerr CE, Jacobson EE (2008). Components of placebo effect: randomised controlled trial in patients with irritable bowel syndrome. BMJ.

[21] Lembo AJ, Conboy L, Kelley JM, Schnyer RS, McManus CA, Quilty MT (2009). A treatment trial of acupuncture in IBS patients. Am J Gastroenterol.

[22] Lee H, Bang H, Kim Y, Park J, Lee S, Lee H (2011). Non-penetrating sham needle, is it an adequate sham control in acupuncture research?. Complement Ther Med..

[23] Vickers A, Goyal N, Harland R, Rees R (1998). Do certain countries produce only positive results? A systematic review of controlled trials. Control Clin Trials.

[24] American Medical Association (2014). Code of medical ethics, 2014–2015: of the American Medical Association.

[25] Emanuel EJ, Miller FG (2001). The ethics of placebo-controlled trials — a middle ground. New Engl J Med.

[26] Wells RE, Kaptchuk TJ (2012). To tell the truth, the whole truth, may do patients harm: the problem of the nocebo effect for informed consent. Am J Bioeth.

[27] Tan K, Petrie KJ, Faasse K, Bolland MJ, Grey A (2014). Unhelpful information about adverse drug reactions. BMJ.

[28] Beardsley E, Jefford M, Mileshkin L (2007). Longer consent forms for clinical trials compromise patient understanding: so why are they lengthening?. J Clin Oncol.

[29] Garner M, Ning Z, Francis J (2012). A framework for the evaluation of patient information leaflets. Health Expect.

[30] Gillies K, Huang W, Skea Z, Brehaut J, Cotton S (2014). Patient information leaflets (PILs) for UK randomised controlled trials: a feasibility study exploring whether they contain information to support decision making about trial participation. Trials.

[31] Wilson CB (2013). An updated declaration of Helsinki will provide more protection. Nat Med.

[32] Dhai A (2014). The research ethics evolution: from Nuremberg to Helsinki. S Afr Med J.

[33] Djavadkhani Y, Marshall NS, D'Rozario AL, Crawford MR, Yee BJ, Grunstein RR (2015). Ethics, consent and blinding: lessons from a placebo/sham controlled CPAP crossover trial. Thorax.

[34] Cheung F (2011). TCM: made in China. Nature.

[35] Park HL, Lee HS, Shin BC, Liu JP, Shang Q, Yamashita H (2012). Traditional medicine in China, Korea, and Japan: a brief introduction and comparison. Evid Based Complement Altern Med..

[36] Volinn E, Yang B, He J, Sheng X, Ying J, Zuo Y (2013). Do outcomes of acupuncture for back pain differ according to varying sociocultural contexts? The view from China. J Altern Complement Med.

[37] Dilli CR, Childs R, Berk J, Christian MK, Nguyen N, Brown RP (2014). Does prior acupuncture exposure affect perception of blinded real or sham acupuncture. Acupunct Med.

[38] White P, Lewith G, Hopwood V, Prescott P (2003). The placebo needle, is it a valid and convincing placebo for use in acupuncture trials? A randomised, single-blind, cross-over pilot trial. Pain.

[39] Hróbjartsson A, Gøtzsche PC (2010). Placebo interventions for all clinical conditions. Cochrane Database Syst Rev.

[40] Kam-Hansen S, Jakubowski M, Kelley JM, Kirsch I, Hoaglin DC, Kaptchuk TJ (2014). Altered placebo and drug labeling changes the outcome of episodic migraine attacks. Sci Transl Med.

[41] Wood L, Egger M, Gluud LL, Schulz KF, Jüni P, Altman DG (2008). Empirical evidence of bias in treatment effect estimates in controlled trials with different interventions and outcomes: meta-epidemiological study. BMJ.

[42] Linde K, Witt CM, Streng A, Weidenhammer W, Wagenpfeil S, Brinkhaus B (2007). The impact of patient expectations on outcomes in four randomized controlled trials of acupuncture in patients with chronic pain. Pain.

[43] Witt CM, Martins F, Willich SN, Schützler L (2012). Can I help you? Physicians' expectations as predictor for treatment outcome. Eur J Pain.

[44] Miller FG, Emanuel EJ, Rosenstein DL, Straus SE (2004). Ethical issues concerning research in complementary and alternative medicine. JAMA.

[45] Menoni V, Lucas N, Leforestier JF, Dimet J, Doz F, Chatellier G (2010). The readability of information and consent forms in clinical research in France. PLoS One.

[46] Reinert C, Kremmler L, Burock S, Bogdahn U, Wick W, Gleiter CH (2014). Quantitative and qualitative analysis of study-related patient information sheets in randomised neuro-oncology phase III-trials. Eur J Cancer.

[47] Srnka KJ, Koeszegi ST (2007). From words to numbers: how to transform qualitative data into meaningful quantitative results. Schmalenbach Business Rev.

[48] Myers MG, Cairns JA, Singer J (1987). The consent form as a possible cause of side effects. Clin Pharmacol Ther.

[49] Pildal J, Hróbjartsson A, Jørgensen KJ, Hilden J, Altman DG, Gøtzsche PC (2007). Impact of allocation concealment on conclusions drawn from meta-analyses of randomized trials. Int J Epidemiol.

