# Supplementary material for: Does different information disclosure on placebo control affect blinding and trial outcomes? A case study of participant information leaflets of randomized placebo-controlled trials of acupuncture
Source: BMC Med Res Methodol. 2018 Jan 18;18:13. doi: 10.1186/s12874-018-0474-1 (PMC5774116; doi:10.1186/s12874-018-0474-1)
Supplement: Additional file 1: — Appendix 1. Demographics of collected PILs. Appendix 2. Placebo control groups in the included studies. Appendix 3. Characteristics of the included studies. Appendix 4. Description of placebo acupunctures in PILs. Appendix 5. How different regions describe placebo acupuncture differently. Appendix 6. A post-hoc subgroup analysis between studies from Asian and non-Asian countries. (DOCX 3144 kb) [file 12874_2018_474_MOESM1_ESM.docx]

**A**

**B**

Appendix 1. Demographics of collected PILs

1. List of included countries with number of PILs. Two additional PILs provided by one author are not counted in this figure. (B) Language distribution among collected PILs. PIL, participant information leaflet.

**Appendix 2. Placebo control groups in the included studies**

Of the 65 included studies, 50 studies used a single placebo control group, and the other 15 studies used more than one control group. In those 15 studies, four studies used two or three different types of placebo acupuncture groups whereas other studies used conventional treatment or waitlist group as additional control groups to the placebo acupuncture control group. Types of placebo acupuncture implemented in the included studies were diverse. They could be divided into three groups in a simple categorization; non-penetrating placebo acupuncture, penetrating placebo acupuncture, and others. Non-penetrating placebo acupuncture was applied at either acupoints (16 control groups) or non-acupoints (19 control groups). Penetrating placebo acupuncture was further divided into either needling at non-acupoints (25 control groups) or at acupoints that are known to be irrelevant with the target disease (six control groups). Other types of placebo control groups included inactive electric stimulation (one control group) and inactive laser acupuncture (four control groups) (Appendix 3).

Appendix 3. Characteristics of the included studies

| **Author (year)** | **Country** | **Sample size** | **Condition** | **Acupuncture** | **Placebo control** |
| --- | --- | --- | --- | --- | --- |
| ***1.1 Non-penetrating placebo at acupoints*** | | | | | |
| Chae et al. 2011a | Korea | 29 | Smokers | MA | Non-penetrating placebo at acupoints |
| Chae et al. 2011b | Korea | 14 | Healthy volunteers | MA | Non-penetrating placebo at acupoints |
| Chou et al. 2011 | Taiwan | 45 | Shoulder pain | MA | Non-penetrating placebo at acupoints |
| Kennedy et al. 2008 | UK | 48 | Acute low back pain | MA | Non-penetrating placebo at acupoints |
| Kleinhenz et al. 1999 | Germany | 52 | Rotator cuff tendinitis | MA | Non-penetrating placebo at acupoints |
| Kreiner et al. 2010 | Uruguay | 32 | Healthy volunteers | MA | Non-penetrating placebo at acupoints |
| Lee et al. 2011 | Korea | 80 | Healthy volunteers | MA | Non-penetrating placebo at acupoints |
| Rebhorn et al. 2012 | Germany | 50 | Healthy volunteers | MA | Non-penetrating placebo at acupoints |
| Schliessbach et al. 2011^a^ | Switzerland | 45 | Healthy volunteers | MA | Non-penetrating placebo at acupoints |
| So et al. 2009 | Hong Kong | 370 | In vitro fertilization | MA | Non-penetrating placebo at acupoints |
| Schliessbach et al. 2012 | Switzerland | 45 | Healthy volunteers | MA | 1) Non-penetrating placebo at acupoints 2) Cold-pressor-induced DNIC |
| White et al. 2012^b^ | UK | 221 | Osteoarthritis | MA | 1) Non-penetrating placebo at acupoints 2) Mock TENS at acupoints |
| Ma et al. 2011 | China | 350 | Labor pain | EA | 1) Non-penetrating placebo at acupoints/E- 2) Conventional care |
| Chung et al. 2012 | Hong Kong | 20 | Postpartum depression | EA | Non-penetrating placebo at acupoints/E+ |
| ***1.2 Non-penetrating placebo at non-acupoints*** | | | | | |
| Bao et al. 2013 | USA | 51 | Musculoskeletal symptoms in breast cancer patients | MA | Non-penetrating placebo at non-acupoints |
| Cho et al. 2013 | Korea | 130 | Chronic low back pain | MA | Non-penetrating placebo at non-acupoints |
| Deng et al. 2013 | USA | 101 | Post-chemotherapy chronic fatigue | MA | Non-penetrating placebo at non-acupoints |
| Harris et al. 2009 | USA | 20 | Fibromyalgia | MA | Non-penetrating placebo at non-acupoints |
| Lao et al. 1999 | USA | 39 | Postoperative pain | MA | Non-penetrating placebo at non-acupoints |
| Lembo et al. 2009^c^ | USA | 262 | Irritable bowel syndrome | MA | Non-penetrating placebo at non-acupoints |
| Paulson and Shay 2013 | Canada | 36 | Healthy volunteers | MA | Non-penetrating placebo at non-acupoints |
| Shen and Goddard 2007 | USA | 15 | Myofascial pain | MA | Non-penetrating placebo at non-acupoints |
| Smith et al. 2011 | Australia | 92 | Primary dysmenorrhea | MA | Non-penetrating placebo at non-acupoints |
| Streitberger et al. 2008 | Germany | 20 | Healthy volunteers | MA | Non-penetrating placebo at non-acupoints |
| Streitberger et al. 2004 | Germany | 220 | Postoperative nausea and vomiting | MA | Non-penetrating placebo at non-acupoints |
| Wayne et al. 2008 | USA | 18 | Chronic pelvic pain | MA | Non-penetrating placebo at non-acupoints |
| White et al. 2000 | UK | 50 | Tension-type headache | MA | Non-penetrating placebo at non-acupoints |
| White et al. 1996 | UK | 50 | Tension-type headache | MA | Non-penetrating placebo at non-acupoints |
| Meng et al. 2012 | China | 23 | Radiation-induced xerostomia | MA and one PA^d^ | Non-penetrating placebo at non-acupoints and one MA |
| Pastore et al. 2011 | USA | 96 | Polycystic ovary syndrome | MA and EA | Non-penetrating placebo at non-acupoints/E- |
| Smith et al. 2013 | Australia | 30 | Fatigue post breast cancer treatment | MA | 1) Non-penetrating placebo at non-acupoints 2) Waitlist |
| ***2.1 Penetrating placebo at irrelevant acupoints*** | | | | | |
| Alecrim-Andrade et al. 2008 | Brazil | 37 | Migraine prophylaxis | MA | Penetrating placebo at irrelevant acupoints |
| Alecrim-Andrade et al. 2006 | Brazil | 28 | Migraine prophylaxis | MA | Penetrating placebo at irrelevant acupoints |
| Hachul et al. 2013 | Brazil | 18 | Insomnia in postmenopause | MA | Penetrating placebo at irrelevant acupoints |
| Guo et al. 2013 | China | 180 | Insomnia | MA and placebo pill | 1) Penetrating placebo at irrelevant acupoints and active pill 2) Penetrating placebo at irrelevant acupoints and placebo pill |
| Vas et al. 2012 | Spain | 275 | Acute low back pain | MA | 1) Penetrating placebo at irrelevant acupoints and conventional care 2) Non-penetrating placebo at non-acupoints and conventional care 3) conventional care only |
| Assefi et al. 2005 | USA | 96 | Fibromyalgia | MA | 1) Penetrating placebo at irrelevant acupoints 2) Penetrating placebo at non-acupoints 3) Non-penetrating placebo at acupoints |
| ***2.2 Penetrating placebo at non-acupoints*** | | | | | |
| Brinkhaus et al. 2006 | Germany | 298 | Chronic low back pain | MA | Penetrating placebo at non-acupoints |
| Jeon et al. 2012 | Korea | 33 | Tinnitus | MA | Penetrating placebo at non-acupoints |
| Kim et al. 2012 | Korea | 33 | Hypertension | MA | Penetrating placebo at non-acupoints |
| Kim et al. 2011 | Korea | 54 | Hot flushes in peri/postmenopause | MA | Penetrating placebo at non-acupoints |
| Li et al. 2012 | China | 75 | Insomnia in heroin addicts | MA | Penetrating placebo at non-acupoints |
| Liang et al. 2011 | China | 190 | Chronic neck pain | MA | Penetrating placebo at non-acupoints |
| Linde et al. 2005 | Germany | 302 | Migraine | MA | Penetrating placebo at non-acupoints |
| Liu et al. 2012^e^ | China | 41 | Myopia | MA | Penetrating placebo at non-acupoints |
| Micalos and Pak 2011 | Australia | 12 | Healthy volunteers | MA | Penetrating placebo at non-acupoints |
| Nedeljkovic et al. 2013 | Switzerland | 20 | Hot flushes in postmenopause | MA | Penetrating placebo at non-acupoints |
| Shin et al. 2013 | Korea | 23 | Healthy volunteers | MA | Penetrating placebo at non-acupoints |
| Shin et al. 2010 | Korea | 130 | Dry eye | MA | Penetrating placebo at non-acupoints |
| Zaslawski et al. 1997 | Australia | 64 | Stress | MA | Penetrating placebo at non-acupoints |
| Wang et al. 2011 | China | 140 | Migraine | MA and placebo pill | Penetrating placebo at non-acupoints and active pill |
| Brinkhaus et al. 2013 | Germany | 422 | Seasonal allergic rhinitis | MA | 1) Penetrating placebo at non-acupoints 2) Waitlist |
| Choi et al. 2013 | Korea | 238 | Allergic rhinitis | MA | 1) Penetrating placebo at non-acupoints 2) Waitlist |
| Langenbach et al. 2012 | Germany | 50 | Postoperative pain | MA | 1) Penetrating placebo at non-acupoints 2) Conventional care |
| Lomuscio et al. 2011 | Italy | 80 | Atrial fibrillation | MA | 1) Penetrating placebo at non-acupoints 2) Conventional care 3) Waitlist |
| Mackenzie et al. 2011 | UK | 105 | Labor pain | MA^a^ | 1) Penetrating placebo at non-acupoints 2) Waitlist |
| Berman et al. 2004 | USA | 570 | Osteoarthritis | MA | 1) Penetrating placebo at non-acupoints and non-penetrating placebo at acupoints 2) Education |
| Andreescu et al. 2011 | USA | 57 | Major depression | EA | Penetrating placebo at non-acupoints/E- |
| Yeh et al. 2012 | Taiwan | 80 | Postanesthetic shivering | EA | Penetrating placebo at non-acupoints/E- |
| Yeung et al. 2012 | Hong Kong | 78 | Insomnia in depression | EA | 1) Penetrating placebo at non-acupoints/E+ 2) Non-penetrating placebo at non-acupoints/E- |
| Yu and Jones 2013 | Hong Kong | 36 | Healthy volunteers | EA | 1) Penetrating placebo at non-acupoints/E+ 2) Non-penetrating placebo at acupoints/E+ |
| ***3. Others*** | | | | | |
| Bergamaschi et al. 2011 | Italy | 34 | Postural instability | Laser AA | AA with inactive laser |
| Ferreira et al. 2013 | Brazil | 53 | Temporomandibular dysfunction | LA | Inactive LA |
| Radvanska et al. 2011^f^ | Denmark | 31 | Nocturnal enuresis | LA | Inactive LA |
| Hsing et al. 2012 | Brazil | 62 | Chronic ischemic stroke | EA | MA |

Abbreviations: AA, auriculoacupuncture; DNIC, diffuse noxious inhibitory control; EA, electroacupuncture; E-, without electrical stimulation; E+, with electrical stimulation; LA, laser acupuncture; MA, manual acupuncture; PA, placebo acupuncture; TENS, transcutaneous electrical nerve stimulation.

^a^Both acupuncture and placebo groups further divided into manual stimulation group and electrical stimulation group.

^b^A multi-factorial design with three doctors, three groups, and two consultation types.

^c^Both acupuncture and placebo groups further divided into augmented interaction and limited interaction groups.

^d^Non-penetrating placebo acupuncture at acupoints.

^e^Both acupuncture and placebo groups further divided into treatment instruction and non-treatment instruction groups.

^f^Inactive LA group further divided into with or without skin contact.

**Appendix 4. Description of placebo acupunctures in PILs**

While the extent to which PILs describe placebo acupuncture varied greatly, there were also some similarities. Other than two studies (3%) that did not mention placebo acupuncture group in their PILs at all, and one study (1.5%) that had the word ‘placebo’ just once in the title of the document and did not have description regarding the control group in the body of the document, most of the studies (62 PILs, 95.4%) informed the participants of the control group in the part where the document introduces the process of the trial. Forty-six PILs provided the relevant information with the words ‘randomization,’ ‘random,’ ‘randomly’ and/or a phrase like ‘you would have an equal chance of.’ In some cases, the explanations went further to help participants understand what randomization meant (e.g. like flipping a coin, by chance). For example, there were subheadings such as screening, randomization, and clinic visits under the procedure section, and explanation for placebo acupuncture group was presented below randomization:

*“Randomization: Acupuncture or Sham Acupuncture*

*After you join the study, you will be randomly assigned (like a flip of a coin) to one of two groups:*

*Group 1: Treatment with acupuncture*

*Group 2: Treatment with sham (fake) acupuncture”*

Regardless of this similarity in format, a closer examination found there were differences in how much description was given to the participants, and extracted data of 70 placebo control groups from 65 PILs were categorized into three groups. In the following sections, the numbers in the parenthesis refers to the number of control groups and percentage out of 70.

***Full disclosure of placebo acupuncture (FD)***

More than half of the PILs (40, 57.1%) were straightforward to the participants when they were introducing placebo acupuncture control. They specifically used words such as ‘placebo (19),’ ‘sham (14),’ ‘fake (2),’ or ‘inactive (2)’ for participants to understand what kind of control acupuncture group is implemented in the study. There were three more documents that were classified into this category even though they lacked the specific words, since they had other wordings that were considered sufficient enough for participants to perceive the existence of placebo acupuncture. For example, a phrase like ‘without treatment effect,’ which can be easily associated with ‘placebo,’ was used. Twenty-seven PILs gave extra explanation for the control group in addition to the simple presentation of the word ‘sham.’ The explanation could go as specific as this:

*“the control group will receive placebo acupuncture only … the placebo needling will be performed at the same acupoints without penetrating the skin.”*

This type of explanation is a representative sample of FD since in acupuncture treatment, whether or not the needle penetrated the skin is considered to be one of the most important factors for producing specific therapeutic effect of acupuncture. Some PILs indeed explained nature of acupuncture in the introduction part with sentences like “Acupuncture is the Chinese art of healing by inserting needles into the skin,” stating explicitly that needle needs to penetrate the skin. Another factor considered important was the location, i.e., the acupoints. Therefore, trials looking into point specificity described placebo control group as

*“The second group will receive “pretend” acupuncture where the needles will be placed in non-acupuncture points.”*

In this particular example, quotation marks were used to emphasize the word ‘pretend.’ There were six more PILs that implemented similar style, putting quotation marks around words such as, placebo, acupuncture-like placebo, sham, sham acupuncture, or fake.

***Deceptive disclosure of placebo acupuncture (DD)***

In the second group, no words or phrases that clearly indicate placebo acupuncture were used (25, 35.7%). Instead, rather deceptive words such as ‘different (7),’ ‘control (5),’ ‘group 1 or group 2 (2),’ ‘non-traditional (2),’ ‘not typical (2),’ and ‘test (2),’ were presented. One PIL simply said ‘acupuncture treatment,’ and others used phrases like ‘simple acupuncture treatment,’ ‘other types of acupuncture,’ ‘contrast point stimulation,’ or ‘one of the four groups.’ These words themselves project neutral image or somewhat deceitful sense of control group, hence may lead the participants to have no clue that placebo control group is involved in the trial. In this category, 14 PILs had extra explanation for the “control group.” However, the extra explanations did not necessarily mean better informing. For example, some PILs wrote phrases that seemed to deliver the idea that both experimental group and control group will receive treatment that has equivalent treatment effect to each other.

*“Participants are divided into two groups of patients receiving different acupuncture treatments: traditional and non-traditional. Above mentioned acupuncture treatments are equally likely to improve patients’ symptoms.”*

Another PIL also narrated in a similar way with a phrase saying “regardless of which group you are in, you'll get the current best medical services.” In a similar way, the PIL that wrote ‘one of the four groups’ in the beginning further explained about sham acupuncture group as “Needling at places that are not relevant to acupuncture which never the less has proven effectiveness in other studies.” On the other hand, there were some other PILs that tried to provide more information, still in a deceptive way. For example, one PIL wrote “Control group gets different treatment from experimental group, which is a commonly used control method in clinical trials of acupuncture.” These types of information seem to lack adequate disclosure since they do not try to convince participants that the control group has treatment effect nor come out clearly that placebo acupuncture will be used, but just portray that it is a control group that differs from the experimental group. Two PILs from Korea and China had similar description of control group using neutral term to name the control group and then explaining in the following sentence that the needle will be placed at non-acupoint. When used with forthcoming terms like sham or placebo, such explanation can be a good additional information distinguishing characteristics of real acupuncture group and placebo acupuncture group. However, by itself, it is likely that it is not enough information for participants to bring to mind that it means a placebo acupuncture group, so the documents were sorted into DD category.

***Missing information on placebo acupuncture (MI)***

Five PILs (7.1%) missed out on explaining anything about placebo control group. In three cases, the whole part where they explain what kind of procedure will be delivered was absent. As a result, not only information on placebo control group, but also on experimental group was missing. For example, one study had a cross-over design with either real or placebo acupuncture in a random order. However, there was no such explanation in the PIL. Instead, only on the questionnaire for the participants (provided by the author), it was written that "new type of acupuncture" and "existing acupuncture" will be compared, and then asked how "first type of acupuncture" and "second type of acupuncture" felt like, which are still words that can easily lead participants to believe that both types of acupuncture are real. Aside from these three studies, there were two studies with certain amount of information on their PILs. One study explained the procedure in detail without clear distinction of experimental group and control group. Instead, there was a sentence at the end mentioning “The acupoint on the skin will either be penetrated or stimulated with just a small pressure.” In the other trial with the double dummy design, the information on both placebo acupuncture and placebo pill was not provided, and the PIL finished the sentence with words assuring that both groups are subject to the equivalent treatment effect.

*“In this study, you will be randomly assigned to either acupuncture or drug treatment groups, but regardless of which group you'll get effective medical services.” ­­­­*

**Appendix 5. How different regions describe placebo acupuncture differently**

After the final categorization of information disclosure in PILs, another grouping of PILs by country of its origin was conducted to explore any potential difference between studies from Asian countries and non-Asian countries. Based on the previous studies that reported expectation and needle sensation might vary and lead to different outcomes [1, 2], we hypothesized that researchers in Asian countries where people are culturally more familiar with acupuncture than non-Asian countries may introduce less or deceitful information on placebo acupuncture for fear of blinding failure.

From the original paper and PIL, information on country where the trial was conducted was extracted, then coded on MS Excel spreadsheets. The country where the document originated from was further categorized into either Asia or non-Asia, reflecting cultural and historical exposure to acupuncture. With this classification, differences in the proportion of categories for PILs between Asian and non-Asian studies were assessed using the chi-squared test. In addition to two-by-three analysis comparing different proportion of each category between Asian and non-Asian studies, three more sensitivity analyses were carried out: for two-by-two crosstabs, three categories of placebo group explanation were grouped into “disclosure (Full Disclosure, FD) vs. no disclosure (Deceptive Disclosure, DD and Missing Information, MI)” or “explanation (FD and DD) vs. no explanation (MI).” Lastly, a comparison between just FD and DD was conducted. For this comparison, 70 placebo groups from 65 PILs were analyzed with chi-squared test or Fisher’s exact test according to the size of variables in a cell. All statistical analyses in this study were done with SPSS (IBM Corp., Version 21.0, Armonk, NY), and the statistical significance was set at P < 0.05.

In the first and second analyses, the number of documents for MI group in both Asia and non-Asia were smaller then 5, so Fisher’s exact test was conducted. The first analysis included all three categories of documents as independent group, and the result showed that how placebo acupuncture is described in a document is associated with its origin of region, revealing that Asian studies being less candid on nature of placebo acupuncture (P = 0.011). The second analysis combined FD and DD into one group to represent PILs with some kind of explanation for placebo control group whereas MI group did not mention anything about placebo. Although documents from non-Asia had lower proportion of documents without explanation than documents from Asia, the difference was not statistically significant (P = 0.051). The third analysis was conducted with groups being divided into FD or the other. Every cell had values higher than 5 since DD and MI groups were combined, so chi-squared test could be used. In this analysis, the result showed documents from non-Asia fully disclosed the existence of placebo acupuncture more than Asian documents did (χ^2^ (1, N = 70) = 7.099, P = 0.008). Lastly, a simple comparison of FD and DD groups also detected a significant difference that is similar to the first and third analyses (P = 0.032). The analysis showed that PILs originating from Non-Asian countries were categorized into FD more than those from Asian countries, meaning more disclosure of information to the participants.

**A**


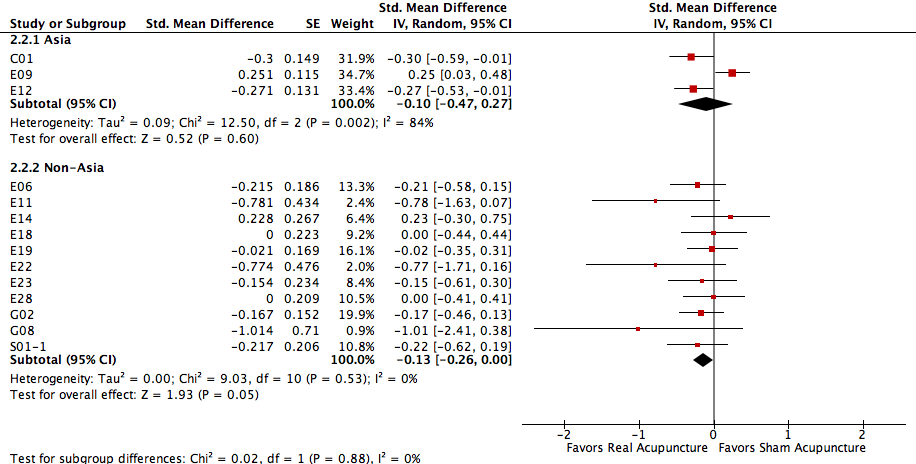


**B**


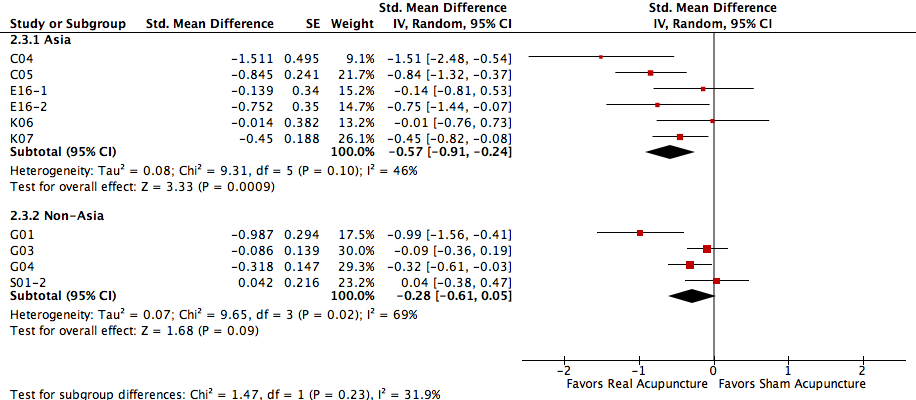


**Appendix 6. A post-hoc subgroup analysis between studies from Asian and non-Asian countries**

1. Asian and non-Asian studies in the FD category. (B) Asian and non-Asian studies in the DD category. DD, deceptive disclosure; FD, full disclosure.

**References**

1. Kalauokalani D, Cherkin DC, Sherman KJ, Koepsell TD, Deyo RA: **Lessons from a trial of acupuncture and massage for low back pain: patient expectations and treatment effects**. Spine*.* 2001;**26**(13):1418-24.

2. Hui KK, Sporko TN, Vangel MG, Li M, Fang J, Lao L: **Perception of Deqi by Chinese and American acupuncturists: a pilot survey**. Chin Med. 2011;**6**(1):2.
